# High throughput clinical CMR service: role of technologists in LV volume analysis

**DOI:** 10.1186/1532-429X-14-S1-P44

**Published:** 2012-02-01

**Authors:** Elisa McAlindon, Chris Lawton, Cornelius Imobeke, Jessica Harris, Barney Reeves, Chiara Bucciarelli-Ducci

**Affiliations:** 1 Bristol Heart Institute, NIHR Cardiovascular BRU, Bristol, UK

## Summary

This study investigates the role of CMR technologists in performing LV volume analysis in a busy clinical CMR service.

## Background

Cardiac magnetic resonance (CMR) imaging studies are increasingly being used in clinical practice for assessment of viability following an acute coronary syndrome. There have been no previous studies investigating the role of CMR technologists in contributing to the analysis of these scans using semi-automated software.

## Methods

20 CMR studies of acute coronary syndromes were included in this study. Analysis was conducted using Argus, Siemens semi-automated volume analysis software. 3 observers conducted the image analysis with two levels of training: Observer 1) “Reference” CMR level 3 physician; Observer 2) and 3) CMR technologists with no previous experience on CMR analysis. Both technologists underwent a 2h tutorial on how to use the software. All 3 observers analysed 20 studies, and re-analysed 10 studies 24 hours after.

## Results

Intra-observer variability was assessed using intraclass correlation coefficient (ICC); inter-observer variability was assessed using Bland Altman plots for agreement.

We observed a good inter-observer variability between Observer 1 and 2, and between Observer 1 and 3 in assessing EDV, ESV, and LV mass (Table 1). Among these parameters, the lowest inter-observer variability was observed for ESV. Bland Altman plots suggested acceptable agreement.

**Table 1:** Inter-observer variability

	Observer	EDV	ESV*	Mass
**1 vs 2**	Mean (SD) difference	15.1 (10.5)	0.1 (0.2)	20.6 (14.2)
	Mean (SD)	154.7 (31.4)	4.3 (0.4)	140.6 (29.2)
	Coefficient of variability (%)	6.8	4.1	10.1

**1 vs 3**	Mean (SD) difference	17.4 (10.9)	0.4 (0.2)	25.6 (14.1)
	Mean (SD)	155.9 (31.3)	4.4 (0.4)	144.5 (30.0)
	Coefficient of variability (%)	7.0	4.7	9.8

Summary statistics calculated are mean and SD of differences, mean and SD of values. Differences between observers are assessed using Bland-Altman plots. *log-transformed for skewed distribution.

Technologist intra-observer variability was comparable to that of the reference observer (ICC technologist volumes and mass 0.94-0.99, ICC reference 0.97-0.99).

## Conclusions

Following a short training, CMR technologists can perform an accurate analysis of LV volume using semi-automated software, compared to an expert CMR physician. These results suggest that technologists can play a role in assisting clinical reporting in a busy clinical CMR service.

## Funding

NIHR Cardiovascular BRU, Bristol Heart Institute.

